# Gut microbiota pattern in children and adolescents with newly diagnosed immune thrombocytopenia

**DOI:** 10.1186/s12887-025-05878-0

**Published:** 2025-07-07

**Authors:** Mohsen Elalfy, Nayra Mehanna, Heba Ghazala, Marwa Tolba

**Affiliations:** 1 Pediatric Hematology Oncology and BMT Department, Faculty of Medicine, Ain Shams University, Cairo, Egypt; 2 Dairy Science and Probiotic Department, Central Laboratories Network, National Research Center, Cairo, Egypt

**Keywords:** ITP, Gut microbiota, Prognosis

## Abstract

**Background:**

Many autoimmune diseases pathophysiology are linked to gut microbiota alteration. We investigated the role of microbiota in newly diagnosed Immune Thrombocytopenia (N-ITP) to determine microbiota changes and its influence on disease course.

**Methods:**

Fifty children with N-ITP (patient group) and 30 control were recruited. 7 microbiota genera were measured in stool samples by real time PCR during the 1st week of presentation before therapy. Bleeding assessment tool and complete blood count (CBC) were performed at enrollment and after 1 week, 1month and 3 months follow-up period.

**Results:**

Early remission occurred in more than 70% of patients. Three strains were isolated only from N-ITP cases and were none in control group. *Bifidobacterium spp*. Detected at a lower rate in patient group compared to control group, but significantly higher in patients progressing to persistent ITP (P-ITP) than patients showing early remission. *Phascolarctobacterium* and *Lactobacillus* detected at significant high rate in N-ITP group compared to control group. Those who show early remission had higher detected level of *Phascolarctobacterium* than patients progressing to P-ITP. *Lachnospiracceae* was detected only in N-ITP who showed early remission. *Bacteroides* was not detected neither in patients nor control.

**Conclusion:**

Gut microbiota behave in different pattern in children with N-ITP and this behavior seems to influence the disease course and may have an impact on future adjunct ITP therapy.

## Introduction

Immune thrombocytopenia (ITP) is an acquired immune-mediated disorder, resulting from peripheral platelet destruction with impaired platelet production [1]. It is defined by isolated thrombocytopenia, a peripheral blood platelet count of < 100 × 10^9^/L, and no obvious underlying cause [2]. The disease has an estimated incidence of 1.9–6.4/100,000 per year in the pediatric population [3]. ITP in children is usually mild with spontaneous resolution 6–18 months after diagnosis in most of patients. However, 20–30% of cases develop chronic ITP [4]. In Egypt, Khalifa and colleagues reported a rate of 30% for cases with chronic ITP in a study taking place in Egypt, which is the same rate reported internationally [5]. Although being mild in children, children and parents are often concerned with the quality of life and changes in some daily activities which highlight disease importance [6]. ITP is classified into newly diagnosed, persistent, or chronic according to the time elapsed from diagnosis: Newly diagnosed is defined from diagnosis till three months. Persistent for disease lasting for three to twelve months from diagnosis and Chronic for disease lasting for more than twelve months [7]. Many factors are considered to decide if the child with ITP will receive medical treatment or be put on watchful waiting and not only the platelet count, especially bleeding symptoms, any recent trauma and child`s lifestyle [8]. Various treatment modalities are recommended according to degree of bleeding. They either increase platelets production or stop the immune mediated platelets destruction [9]. Newer treatment modalities could be suggested based on better understanding of ITP pathophysiology. Although the pathogenesis of ITP is incompletely understood, ITP is considered a complex multifactorial immune dysregulation in which the primary mechanism is the loss of immune tolerance, and platelet autoantibodies production [10]. Also, immunologic mechanisms of T cells have also been suggested to cause ITP. This includes T cell-mediated cytotoxicity and defects in regulatory T cells (T-Regs) function and/or number [11, 12]. Gut microbiota has been suggested to play a role in ITP pathogenesis. It refers to a community of microorganisms living naturally in humans` digestive tracts [13]. They include bacteria, viruses, and some eukaryotes that colonize digestive tract just after birth [14]. Humans have their own individual pattern of microbiota distribution and composition, which is in part determined by the host genotype and by the colonization occurring immediately after birth. Many factors determine the individual microbiota pattern including mode of delivery, breastfeeding, lifestyle, diet, environmental conditions, antibiotic use, and vaccination [15].

The intestinal microbiota has more than 1500 species, formed of more than 50 different phyla [16]. The most dominant phyla in humans are *Bacteroidetes* and *Firmicutes* followed by *Proteobacteria*,* Fusobacteria*,* Tenericutes*, *Actinobacteria* and *Verrucomicrobia* making up 90% of the total microbial population [17]. Intestinal microbiota plays an important role on the immune response of humans. It is crucial for development and expansion of lymphoid tissues as well as maintenance and regulation of intestinal immunity [18, 19]. Commensal microbiota act as the outside-in modifier of T cell and natural killer cells subsets. They maintain the integrity of the mucosal barrier [20, 21]. T cells can generate subpopulations of which immune responses may be anti or pro-inflammatory. *Bacteroides* fragilis is capable of inducing differentiation of CD4 + T cells into regulatory T cells, favouring the production of anti-inflammatory cells and the transforming growth factor beta (TGF-β), thus neutralizing the pro-inflammatory response of That (T helper). On the other hand, the group of filamentous bacteria, after contacting the antigen-regulating cells, seems capable of inducing pro-inflammatory cells such as Th17 [22].

Different factors result in loss of beneficial microbes and a reduction in microbial diversity, ultimately triggering gut dysbiosis (microbial imbalance or maladaptation) [23]. Dysbiosis can alter the immune response, thus promoting a pro-inflammatory state. Such alterations, can originate or favor the onset of several diseases [24]. Many autoimmune diseases have been linked to gut microbiota perturbation including Rheumatoid Arthritis, Multiple Sclerosis, Systemic Lupus Erythematosus, Autoimmune Liver Disease, Graves‘s disease, skin related autoimmune pathologies, and Inflammatory Bowel Disease [25–27]. ITP, being an immune mediated disease, was also linked to gut microbiota alteration. *Borody et al.* [28] reported that fecal microbiota transplantation successfully reversed ITP. Previous studies have been inconsistent with intestinal microbiota pattern in ITP, with a more significant percentage of Proteobacteria and *Bacteroidetes* and a lower ratio of *Firmicutes/Bacteroidetes* in ITP compared with healthy controls, others showed that *Actinobacteria* and the *Firmicutes/Bacteroidetes* ratio decreased, while the opposite *Firmicutes/Bacteroidetes* ratio had been reported [29–31]. Moreover, gut microbiota and the metabolome of patients with ITP showed metabolic changes. *Saki et al.* showed that specific phyla and genera undergo the most notable changes in patients with ITP. Furthermore, they demonstrated changes in various gut metabolites, including lipids, amino acids, and bile acids in patients with ITP [32]. Strong negative correlation was reported between platelet counts and intestinal microbiota, as well as between platelet counts and intestinal metabolites [33]. Also dietary yeast plays an important role in ITP by changing the composition of intestinal flora and generating many advantageous cytokines in individuals with ITP [34].

Our objective was to assess gut microbiota alteration among children with newly diagnosed ITP (N-ITP) and to determine its relation to the disease course and bleeding score. To our knowledge, this is the first Egyptian study to assess gut microbiota in children and adolescents with ITP.

## Methodology

### Patients

This was a prospective longitudinal case control study recruited children and adolescents with newly diagnosed ITP based on the American Society of Haematology (ASH) definition of ITP [35] during their first week after diagnosis. Eligible children and adolescents aged 2–15 years were recruited from the Paediatric Haematology Ooncology and BMT department, Ain Shams University over 6 months consequently from April to October 2023 before initial treatment modalities [36]. Age and sex matched healthy volunteers were chosen not from the patients` families. Patients and control were excluded if suspected familial or genetically associated ITP, or if they had received antibiotics in the previous 3 months or had a history of acute or chronic gastrointestinal diseases, metabolic syndromes or patients who had a history of bowel surgery or other autoimmune diseases. The study was conducted after approval of the Ethical Committee under acceptance number FMASU MS/000017585. Informed consent and assent were obtained from each patient and control or their legal guardians before enrolment in the study. All procedures performed were in accordance with the ethical standards of the institutional research board in accordance with Helsinki declaration 2013.

### Methods

Upon study entry, all patients and control were subject to Evaluation of different bleeding symptoms using ITP- specific bleeding assessment tool (ITP-BAT) which was supported by the international working group of ITP to be used in further clinical studies. It consists of three main domains: skin (S), visible mucosae (M), and organs (O), including grading of severity (SMOG). Severity ranges from 0 to 3 or 4, with grade 5 given to any fatal bleeding. Bleeding that was reported by the patient without medical documentation is graded 1 [37]. Complete blood picture (CBC) with manual platelet count and assessment of the proportion of seven specific microbiota as *Bifidobacterium spp*,* Bacteroides*, *Phascolarctobacterium*,* Lactobacillus*,* Ruminococcaceae (A bacterial family that include Ruminococcus and other bacteria)*,* Eubacterium coprostanoligeues and Lachnospiraceae* in stool samples of all participants was done by using the real time Polymerase Chain Reaction (PCR). The choice of the microbiota genus to be evaluated in our study was based on the previous study done by Yu and colleagues that demonstrated significant variation in these microbiota genus proportions between healthy controls and newly diagnosed ITP but in adults [30]. Stool specimens were collected, kept in sterilized caps at + 4°c in the refrigerator and delivered to PCR laboratory within 4 h for DNA extraction. The amount of genomic DNA extracted was determined by Nanodrop measurements to determine the DNA quality [38]. The primers and probes used to detect *Bifidobacteria*,* Streptococci*, and *Lactobacillus* species were based on 16 S rRNA gene sequences retrieved from the National Center for Biotechnology Information databases [39]. For the real-time PCR experiments, the DNA was combined with Power SYBR™ Green PCR Master Mix from Thermo Fisher.

**Fig. 1:**
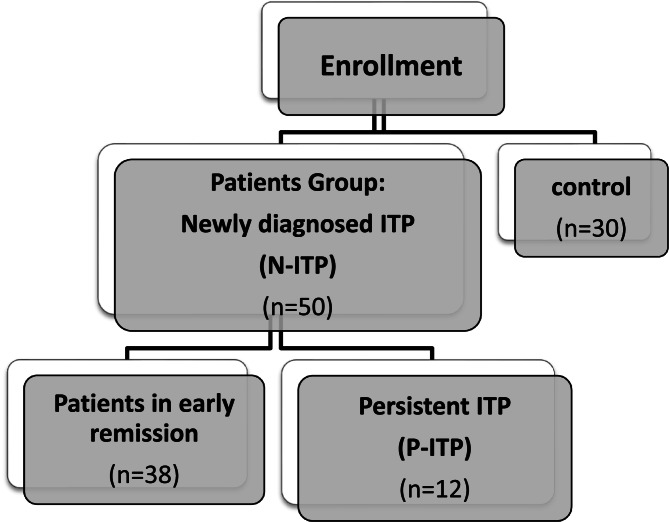
Consort flow diagram for the enrolled patients in the patients group and control group

Patients were followed up regularly every 2 weeks with CBC and ITP-BAT [37], while data collection at one week, one month and three months from study entry. Patients received treatment according to the international consensus report by Provan and colleagues 2019, conservative management if platelet count > 30 10^3^/uL with no active bleed. Steroid therapy: 7 days or shorter courses with prednisone (2–4 mg/kg/day; maximum, 120 mg daily, for 5–7 days). Intravenous immunoglobulins (IVIG) used dose was 1 g/kg on 1 or 2 consecutive days. Eltrombopag was administered at an initial dose of 25 or 50 mg/d, depending on patient age, up to a maximum of 75 mg/day [36]. By the end of the three months, patients were categorized according to their disease course to a subgroup of patients in early remission and a subgroup of patients with Persistent ITP (P-ITP) [7] as shown in (Fig. 1).

#### Sample size

A sample size of 50 patients and 30 sex and age matched healthy controls (HCs) was sufficient to reach the study objective based on a hypothesized proportion of outcome in the population. Sample size was calculated Using PASS 15 program for sample size calculation, setting power at 90% and alfa error at 0.05, and according to *Zhang and colleagues* [31], the expected depletion of bacteriodetes in ITP vs. HCs is 20.21 ± 2.75% vs. 39.49 ± 2.49%.

#### Statistical analysis

Data were collected, revised, coded and entered to the Statistical Package for Social Science (IBM SPSS) version 27. The confidence interval was set to 95% and the margin of error accepted was set to 5%.

## Results

**Fig. 2:**
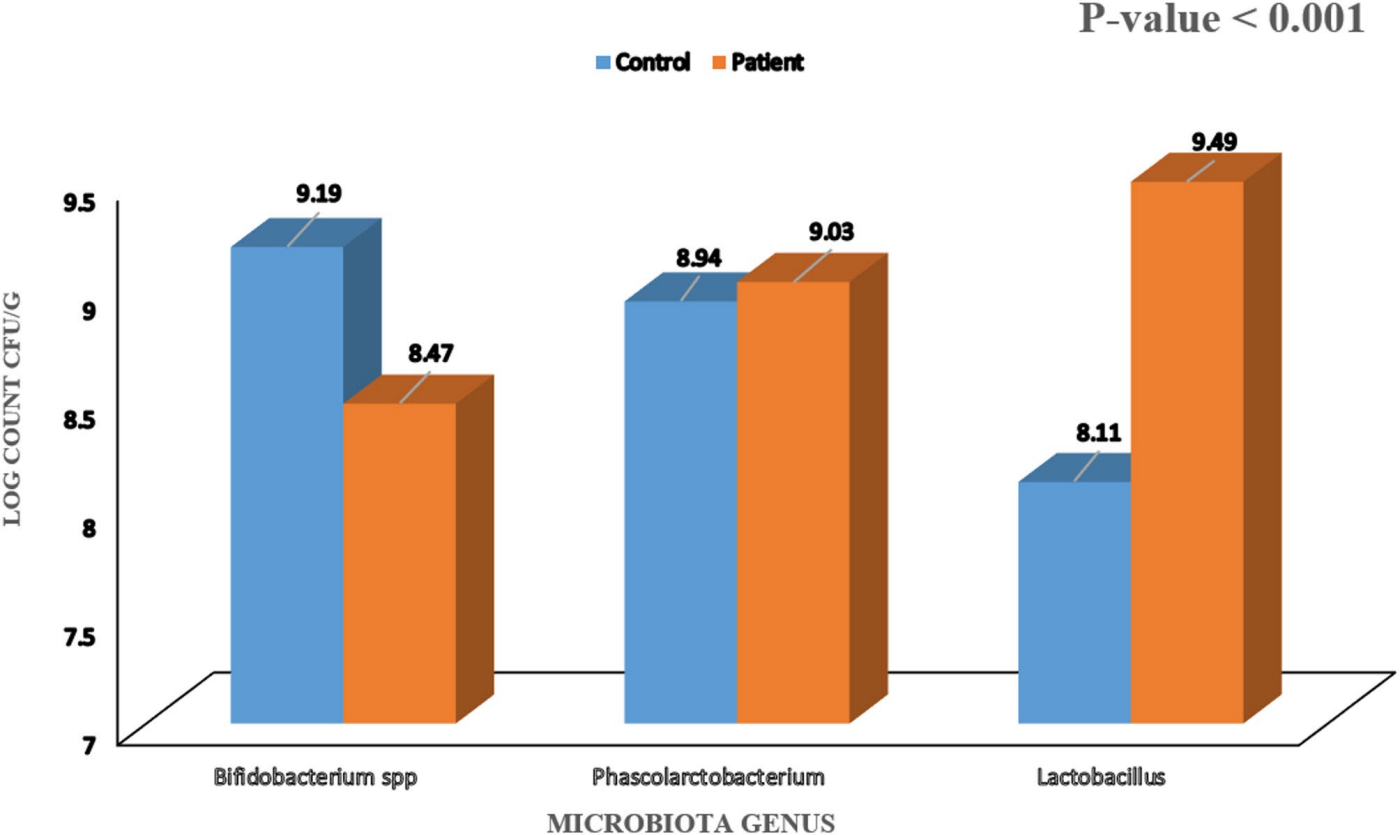
Comparison between control and patients groups regarding Microbiota genera (log count) cfu/g (Upon study entry)

The demographic, bleeding characteristics and therapeutic data of patients are illustrated in Table 1. Their median age was 6 years. By the end of the study period, 38 patients (76%) were in remission while 12 patients (24%) were considered persistent ITP (P-ITP). Patients in the P-ITP group had significantly higher age than patients with early remission with median 11.5 years (8.5–12.5 years) in the P-ITP group compared to 5 years median age (3–8 years) in the early remission group, with no significant difference regarding consanguinity or bleeding scores. All (100%) patients had presented with skin bleeding, 12 (24%) patients had mucosal bleeding and only 4 (8%) patients had organ bleeding. Patients with organ bleeding were adolescent females whose age ranged (12–14 years) and presented with menorrhagia that led to hemoglobin drop with the lowest hemoglobin reaching 7 g/dl. Table 2 showed the laboratory parameters and bleeding score of the patient group upon study entry, at one week, one month and three months (end of study). Comparison between the patient group and control group as regard the microbiota genus as depicted in (Fig. 2) showed that *Ruminococcacae*,* Eubacterium coprostanoligeues and Lachnospiracceae* genus were only detected in patients group and not in control group. *Bifidobacterium spp.* had a higher mean log count in the control group (9.19 ± 0.11) compared to the patient group (8.47 ± 0.15) (*P* < 0.001). *Phascolarctobacterium* genus was significantly higher in the patient group (9.03 ± 0.11) compared to the control group (8.94 ± 0.07) with *P* value < 0.001. Also *Lactobacillus* genus was significantly higher in the patient group (9.49 ± 0.19) compared to the control group (8.11 ± 0.13) with *P*- value < 0.001.

**Table 1:** Demographic characteristics, bleeding score and therapeutic data of all N-ITP patients

Demographic characteristics		Patient group
		No.= 50
Age of onset(years)	Median (IQR)	6 (3 - 11)
	Range	2–15
Sex	Male n (%)	21 (42%)
	Female n (%)	29 (58%)
Final diagnosis at end of study	N-ITP in early remission n (%)	38 (76%)
	Persistent ITP n (%)	12 (24%)
Bleeding score (ITP-BAT)		
Skin (S) n (%)	S0	0 (0%)
	S1	14 (28.0%)
	S2	29 (58.0%)
	S3	7 (14.0%)
Mucous membrane (M) n (%)	M0	38 (76.0%)
	M1	8 (16.0%)
	M2	4 (8.0%)
Organ (O) n (%)	O0	46 (92%)
	O1	1 (2.0%)
	O3	1 (2.0%)
	O4	2 (4.0%)
Patients who received treatment n (%)		45 (90.0%)
Steroids		44 (88.0%)
Start (weeks from diagnosis)	1 week	43 (97.7%)
	2 weeks	1 (2.3%)
Duration (days)	Mean ± SD	6.98 ± 1.92
	Range	3–10
IVIG n (%)		5 (10.0%)
Start (weeks from diagnosis)	Median (IQR)	3 (1 - 4)
	Range	1–5
Frequency of dosing	Once	5 (100%)
Eltrombopag		10 (20.0%)
Start (weeks from diagnosis)	Mean ± SD	6.2 ± 3.01
	Range	4–12
Duration (months)	Mean ± SD	2.65 ± 0.75
	Range	1–3

*IQR *interquartile range, *SD *standard deviation, *N-ITP* newly diagnosed ITP, *ITP-BAT* ITP Bleeding Assessment Tool, *IVIG* intravenous immunoglobulin

**Table 2:** Laboratory parameters and bleeding score of the N-ITP patient upon study entry, at 1 week, 1 month and 3 months follow up

		Upon entry	1 week	1 month	3 months	Test value	*P*-value
CBC							
Lymphocytes 10^3^/uL	Mean ± SD	3.24 ± 1.5	3.52 ± 1.66	3.72 ± 1.7	3.32 ± 1.16	1.49•	0.224
	Range	0.9–7.21	0.82–8.1	1.48–9	1.3–7.2		
Hemoglobin g/dL	Mean ± SD	11.16 ± 1.58	11.5 ± 1.22	11.89 ± 1.11	11.92 ± 1.14	7.53•	0.001
	Range	8–13.8	8.8–14	9.4–14.1	8.1–14.3		
Platelets 10^3^/uL	Median (IQR)	13 (7 - 21)	86 (28 - 195)	198 (99 - 287)	248 (180 - 317)	78.51≠	0.000
	Range	1–65	2–426	15–457	1–792		
Bleeding score ITP-BAT							
Skin (S) n (%)	S0	0 (0%)	34 (68%)	45 (90%)	42 (84%)	146.34*	0.000
	S1	14 (28.0%)	16 (32%)	5 (10%)	8 (16%)		
	S2	29 (58.0%)	0 (0%)	0 (0%)	0 (0%)		
	S3	7 (14.0%)	0 (0%)	0 (0%)	0 (0%)		
Mucosa (M) n (%)	M0	38 (76.0%)	50 (100%)	50 (100%)	50 (100%)	38.30*	0.000
	M1	8 (16.0%)	0 (0%)	0 (0%)	0 (0%)		
	M2	4 (8.0%)	0 (0%)	0 (0%)	0 (0%)		
Organ (O) n (%)	O0	46 (92%)	50 (100%)	50 (100%)	50 (100%)	12.25*	0.199
	O1	1 (2.0%)	0 (0%)	0 (0%)	0 (0%)		
	O3	1 (2.0%)	0 (0%)	0 (0%)	0 (0%)		
	O4	2 (4.0%)	0 (0%)	0 (0%)	0 (0%)		

*IQR* Interquartile Range, *SD *standard deviation, *ITP-BAT *ITP Bleeding Assessment Tool

*• *Repeated Measures ANOVA test, ≠ Friedman test, * Chi-square test

**Fig. 3:**
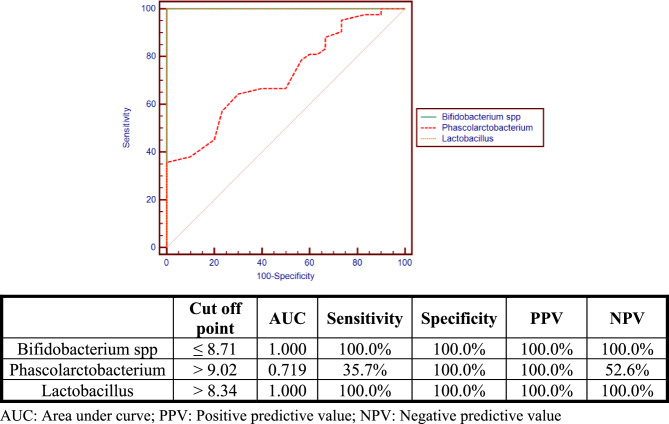
Receiver operating characteristic curve (ROC) curve. It showed that *Bifidobacterium spp. Phascolarctobacterium and Lactobacillus* can differentiate between patients group and control group at the cut off point ≤ 8.71, > 9.02 and > 8.34 respectively with sensitivity 100.0%, specificity 100.0% and AUC of 1.000, 0.719 and 1.000 respectively

Receiver operating characteristic curve (ROC) shown in (Fig. 3) showed that *Bifidobacterium spp. Phascolarctobacterium* and *Lactobacillus* Can differentiate between patients group and control group at the cutoff point ≤ 8.71, > 9.02 and > 8.34 respectively with sensitivity 100.0%, specificity 100.0% and AUC of 1.000, 0.719 and 1.000 respectively. At presentation the comparison of the microbiota pattern between patients who showed early remission and those who had P-ITP showed significant difference as illustrated in Table 3. *Bifidobacterium spp*. had a significantly higher mean log count in the P-ITP group (8.58 ± 0.09) compared to the group with early remission (8.44 ± 0.15) with *p*-value 0.005. *Phascolarctobacterium* showed a significantly higher mean log count in the group with early remission (9.06 ± 0.11) compared to the P-ITP group (8.94 ± 0.06) with *p*-value 0.002. *Eubacterium coprostanoligeues* was also significantly higher in the P-ITP group (9.06 ± 0.1) compared to the group with early remission (8.81 ± 0.16) with *p*-value < 0.001. The univariate logistic regression analysis presented in Table 4 shows that age > 10 years, *Bifidobacterium* > 8.5, *Phascolarctobacterium* < 8.95 and *Eubacterium coprostanoligeues* > 8.96 were significantly associated with patients with P-ITP. Also, the multivariate logistic regression analysis shows that the most important factors associated with patients with P-ITP was *Eubacterium coprostanoligeues* > 8.96 with OR (95%CI) of 61.810 (5.554–687.901) and with *p*-value = 0.001 followed by *Bifidobacterium* > 8.5 with OR (95% CI) of 15.694 (1.458–168.974) and with *p*-value = 0.023.

**Table 3:** Comparison between initial Microbiota strains (log count) cfu/g. in N-ITP with early remission and in persistent ITP by the end of the study period

**Microbiota log count**		**N-ITP in early remission(*N*= 38)**	**Persistent ITP(*N*= 12)**	**Test value**	**Adjusted *P*-value**
		**Median (IQR)**	**Median (IQR)**		
Bifidobacterium spp	Mean ±SD	8.44 ± 0.15	8.58 ± 0.09	−2.974•	0.012
	Range	8.09 - 8.65	8.43 - 8.71		
Bacteroides	Not detected	38 (100.0%)	12 (100.0%)	-	-
Phascolarctobacterium	Mean ±SD	9.06 ± 0.11	8.94 ± 0.06	3.387•	0.009
	Range	8.9 - 9.3	8.85 - 9.01		
Lactobacillus	Mean ±SD	9.46 ± 0.2	9.56 ± 0.14	−1.369•	0.269
	Range	9.09 - 9.87	9.26 - 9.73		
Ruminococcaceae	Mean ±SD	5.57 ± 0.11	5.6 ± 0.16	−0.239•	0.818
	Range	5.47 - 5.68	5.4 - 5.81		
Eubacterium coprostanoligeues	Mean ±SD	8.81 ± 0.16	9.06 ± 0.1	−5.132•	0.009
	Range	8.47 - 9.03	8.89 - 9.17		
Lachnospiraceae	Mean ±SD	5.65 ± 0.14	-	-	-
	Range	5.41 - 5.9	-		

*N-ITP* (Newly diagnosed ITP), *SD *standard deviation, *IQR* interquartile range

• Independent t-test

**Table 4:** Univariate and multivariate logistic regression analysis for factors associated with patients with P-ITP

	**Univariate**				**Multivariate**			
	***P*-value**	**OR**	**95% C.I.for OR**		***P*-value**	**OR**	**95% C.I.for OR**	
			**Lower**	**Upper**			**Lower**	**Upper**
Age > 10 years	0.000	25.500	4.812	135.126	-	-	-	-
Bifidobacterium > 8.5	0.028	5.143	1.190	22.222	0.023	15.694	1.458	168.974
Phascolarctobacterium ≤ 8.95	0.010	6.444	1.567	26.506	-	-	-	-
Eubacterium coprostanoligeues > 8.96	0.000	26.667	4.630	153.573	0.001	61.810	5.554	687.901

*OR* Odds ratio, *CI* Confidence interval

**Fig. 4:**
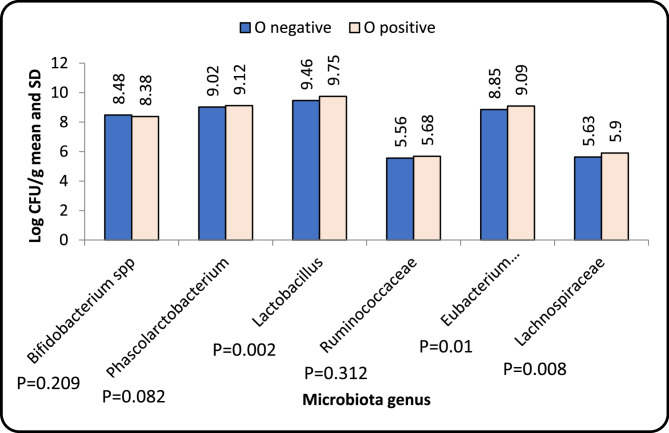
Relation between organ bleeding (O score) and microbiota genus among the studied N-ITP patients upon study entry

Platelet counts of all the studied patients upon study entry did not correlate significantly with the different microbiota genera (*Bifidobacterium spp* r value = 0.14 and *p* value = 0.34, *Phascolarctobacterium* r value= −0.23 and *p* value = 0.14 and *Lactobacillus* r value = 0.009 and *p* value = 0.953). There was no significant correlation between the skin bleeding score (S score) and the mucosa bleeding score (M score) with the studied microbiota genera at study entry with *p*-value > 0.05. The relation between the organ bleeding manifestation (0 score) and the different microbiota genera among the studied patients is presented in (Fig. 4). There was significant increase in the level of *lactobacillus* in patients with positive O score [9.75 ± 0.11] than patients with negative O score [9.46 ± 0.17] with *p*-value = 0.002. Also, the level of *Eubacterium coprostanoligeues* was significantly higher in patients with positive O score [9.09 ± 0.07] than patients with negative O score [8.85 ± 0.18] with *p*-value = 0.012. There was also significant increase in the level of *lachnospiraceae* in patients with positive O score [5.9 ± 0.01] than patients with negative O score [5.63 ± 0.13] with *p*-value = 0.008.

## Discussion

Gut microbiota participates in a variety of important physiological functions of the host including maturation of the immune system [30]. Any imbalance in the interactions between microbiota and immune system plays a role in the pathogenesis of many immune mediated diseases. A recent study demonstrated a link between gut microbiota and ITP [25].

In this study, we enrolled over six months` period 50 young N-ITP patients. At the end of 12 weeks period, 38 patients (76%) N-ITP were in remission, this is considered a high remission rate, however possibly due to short follow-up, some of patients when off-treatment might show thrombocytopenia. Only 12 (24%) had persistent course (P-ITP group). A younger age below 6 years compared to children above 12 years, but neither consanguinity nor bleeding scores had an impact on early remission. All patients had ecchymosis or petechiae, one-quarter had mucous membrane bleeding and only 4 (8%) had organ bleeding. Steroids were given for 88% (*n* = 44) of the patients. 10% (*n* = 5) of the patients received IVIG because of high bleeding score of (S2 or M2), lower PC < 10 × 10^3^/uL and/or poor response to steroid therapy after a week of persistent bleeding symptoms. Eltrombopag was given as a second line therapy after more than 1 month from study entry in 20% of the patients due to failure of response to first line therapy in these patients. Five patients showed spontaneous disease remission without the need of initiation of any platelet enhancing therapy. These five patients were < 6 years, presented only with mild skin bleeding and their platelet counts were around 20 × 10^3^/uL.

A clear difference in the mean log count of the microbiota types was obvious in patient group compared to control in our study. *Oved et al.* [40] concluded similar findings stating that pediatric patients with newly diagnosed ITP have demonstrated gut dysbiosis. Significant differences were observed in studied microbiota as for *Bifidobacterium spp.*, which had a lower mean log count in the patient group compared to the control group. However, *Phascolarctobacterium* and *Lactobacillus* were significantly higher in the patient group compared to the control group. A study by *Li et al.* [39] found similar results. They found an increase in Firmicutes phylum (a phylum to which *Lactobacillus genus* belong) and also decrease in *Actinobacteria* with a significant decrease in *Bifidobacterium spp* in ITP patients. Also a study conducted by *Yu* and colleagues [30]. found that the *Phascolarctobacterium* and *Lactobacillus* were significantly higher in ITP patients. A study on ITP in 30 adults by Zhang et al. who revealed that the variation of microbiota in ITP patients were mainly due to enrichment of *Lactobacillus* which is similar to our study finding [31]. Moreover, in our study, 3 species, *Eubacterium coprostanoligeues*,* Lachnospiraceae* and *Ruminococcaceae* were detected in the ITP cases but not in control, which may need further studies to find out, if linked to a possible role in ITP pathogenesis through mediating inflammation. However, another study reported that *Lachnospiraceae* and *Ruminococcaceae* were found to have a role in immune regulation, their metabolites can induce immune tolerance through T reg cells [41–43]. In contrast to Yu and colleagues 2022 [30] who found that *Bacteroides* were enriched in their ITP patients group, *Bacteroides* were not detected in both our cases and control group despite meticulous work-up, it might be explained by the suggested country wise microbial signatures which highlights the role of environmental factors as well as host genetics in shaping the gut microbiota composition [44].

Moreover, our study revealed that that there was significant difference found between patients who showed early disease remission and those with persistent ITP regarding microbiota pattern at presentation. *Phascolarctobacterium* was significantly higher in patients who showed early remission than those who developed P-ITP. *Phascolarctobacterium* was considered a substantial producer of short chain fatty acids (SCFAs) including acetate and propionate [45]. SCFAs has an important role in immune regulation as they increase the number of T reg cells as well as their activity and inhibit CD4 + cells. They also demonstrated reduction in gut inflammation and enhancing the expression of anti-inflammatory cytokines such as IL-10 [46]. This could explain the significantly higher *Phascolarctobacterium* in patients who showed early remission in our study. Similarly, Wang et al. revealed that there were differences in gut microbiome between N- ITP, persistent ITP and chronic ITP which in part compatible with our study at initial presentation, but with different studied microbiota species [47]. Also our study showed significantly higher levels of *Bifidobacterium* in the P-ITP group compared to those who went into early remission despite being lower in the overall ITP patients compared to controls. Since P-ITP is seen more in adolescents, these microbiota differences between the P-ITP and the early remission group could be attributed to age and sex hormones. Estrogen and testosterone demonstrated a direct effect on immune cells as well as the gut microbiota [48]. In another study, progesterone was suggested to promote *Bifidobacterium* increase in late pregnancy [49]. Thus, the interaction between Gut microbiota, sex hormones and immunity can suggest an explanation to the changes in microbiota between the P-ITP and patients with early remission.These data suggest the possible role of microbiota in determining the course of the disease and response to therapy and hence guide treatment decision.

Although there was significant difference between the microbiota in patients with and without organ bleeding, no direct relation could be found in literature that link bleeding with the different microbiota detected. This maybe related to the inflammatory process and the severity of the disease rather than bleeding itself.

Inspite there was difference in microbiota between our different patient groups initially, follow up of the microbiota distribution was out of the scope of our study and thus, we didn’t study effect of pre or pro biotics on the patients. Altering diet using dietary yeast may assist in future management of ITP by inducing microbiota changes.

### Limitations

This pilot single arm prospective study did not include infants below 2 years. The short duration of follow-up could not really differentiate a group of patients, classified as early responders to progress again to be persistent or chronic ITP [50]. Small number of enrolled patients could be a limitation. The direct relation between the microbiota genus and immune regulatory cells, cytokines or the dynamic change of the microbiota in different phases of the disease was beyond the scope of the study. We didn’t study effect of prebiotics or probiotics on our patients. Limited previous studies on the pattern of gut microbiota in healthy Egyptian children [51]. Effect of treatment on the microbiota was not studied as well. Although studying metabolomics/metagenomics, would be of more value, it needed higher budget and can be done in a future multicenter study.

## Conclusion

Microbiota alteration seems to influence course of the N-ITP. These insights advocate for further large-scale studies to confirm the associations observed between specific gut microbiota and ITP disease course, thus opening the door to explore microbiota-targeted therapy as potential adjunct treatment for ITP in the future.

## Data Availability

The dataets used and/or analysed during the current study are available on reasonable request from the corresponding author.

## Abbreviations

ASH: American Society of Hematology
AUC: Area Under Curve
BMT: Bone Marrow Transplantation
CI: Confidence interval
HB: Hemoglobin
ITP: Immune Thrombocytopenia
ITP-BAT: ITP Bleeding Assessment Tool
IVIG: Intravenous Immunoglobulins
M score: Visible mucosa bleeding score
N-ITP: Newly diagnosed ITP
OR: Odds Ratio
O score: Organ bleeding score
P-ITP: Persistent ITP
PC: Platelet Count
ROC curve: Receiver Operating Characteristic Curve
SCFAs: Short Chain Fatty Acids
S score: Skin bleeding score
SMOG: Skin, Mucosa and organ grading
